# COPING AND LIFE SATISFACTION OF OLDER PEOPLE DURING THE COVID-19 PANDEMIC

**DOI:** 10.1093/geroni/igac059.3134

**Published:** 2022-12-20

**Authors:** Dongjuan Xu, Nasreen Lalani, Gregory Arling

**Affiliations:** Purdue University, West Lafayette, Indiana, United States; Purdue University, West Lafayette, Indiana, United States; Purdue University, West Lafayette, Indiana, United States

## Abstract

Covid-19 put older individuals at high risk for increased morbidity and mortality, isolation, reduced coping and life satisfaction. Optimism, sense of mastery and closeness with family and friends can enhance coping and life satisfaction among older adults. No such studies were found during the pandemic. Our study examined the associations between optimism, sense of mastery, closeness with spouse, family, and friends, physical and psychological functioning and its effects on coping and life satisfaction. A national representative sample of 1,890 community dwelling older adults was obtained from the 2020 Health and Retirement Study COVID-19 data during March 2020-June 2021. A structural equation modeling approach used to test the associations and their direct and indirect effects on life satisfaction. Coping was seen as a mediator affecting these relationships and their effects on life satisfaction. Optimism (β = .318, p < .001), mastery (β = .195, p < .001) closeness with spouse/partner (β = .199, p < .001), closeness with children ((β = .075, p < .010), friends (β = .086, p < .001), had significant positive direct and indirect effects on life satisfaction. Frailty (β = -.137, p < .001), comorbidities (β = -.057, p < .050), and IADL limitations (β = -.118, p < .001) had negative direct effects on life satisfaction. Optimism, sense of mastery and closeness with family/friends promotes coping and life satisfaction whereas frailty and comorbidities negatively influence coping and life satisfaction of the older adults. Community interventions should target coping strategies that enhances optimism, mastery, and interpersonal closeness among older adults during pandemic.

